# Lectures on Clinical Medicine

**Published:** 1874-01

**Authors:** 


### Books Reviewed.

*Lectures on Clinical Medicine.* By A. Trousseau, Late Professor of Clinical Medicine in the Faculty of Medicine, Paris. Translated from the Third Enlarged and Revised Edition. By Sir John Rose Cormack, M. D., F. R. S. E., and P. Victor Bazire, M. D. Complete in Two Volumes. Philadelphia: Lindsay & Blackiston, 1873. Buffalo: T. Butler & Son.

At this time to attempt to give a Review of Trousseaus' Clinical Medicine, would be attempting to inform the profession of the merits of a work with which they are already fully acquainted. The third edition of these lectures have been under the editorial supervision of M. Peter, formerly Trousseau's *chef de clinique*, and it is from this edition that the present translation is made. One of the objections to the former English edition, was the number of volumes which it comprised, but this is overcome by the publishers of the present one who have condensed the five volumes into two. This has been done without any sacrifice of the amount of material which was contained in the five volumes, except the notes of the translators. The lectures are printed in type sufficiently plain to be easily read. The lectures are arranged in the order in which they were delivered by Prof. Trousseau.

Messers. Lindsay and Blakiston, have certainly placed the profession under obligations for this neat and handy edition of Trousseau's Clinical Lectures.

